# Hypertension and Type 2 Diabetes—The Novel Treatment Possibilities

**DOI:** 10.3390/ijms23126500

**Published:** 2022-06-10

**Authors:** Agnieszka Przezak, Weronika Bielka, Andrzej Pawlik

**Affiliations:** Department of Physiology, Pomeranian Medical University in Szczecin, 70-111 Szczecin, Poland; agn-prze@wp.pl (A.P.); weronika.bielka@wp.pl (W.B.)

**Keywords:** hypertension, type 2 diabetes, antihypertensive drugs

## Abstract

Elevated blood pressure and hyperglycaemia frequently coexist and are both components of metabolic syndrome. Enhanced cardiovascular risk is strongly associated with diabetes and the occurrence of hypertension. Both hypertension and type 2 diabetes, if treated inappropriately, lead to serious complications, increasing the mortality of patients and generating much higher costs of health systems. This is why it is of great importance to find the missing link between hypertension and diabetes development and to simultaneously search for drugs influencing these two disorders or even drugs aimed at their pathological bases. Standard antihypertensive therapy mainly focuses on blood pressure reduction, while novel drugs also possess a wide range of pleiotropic modes of actions, such as cardio- and nephroprotective properties or body weight reduction. These properties are especially desirable in a situation when type 2 diabetes coexists with hypertension. This review describes the connections between diabetes and hypertension development and briefly summarises the current knowledge regarding attempts to define targets for the treatment of high blood pressure in diabetic patients. It also describes the standard hypotensive drugs preferred in patients with type 2 diabetes, as well as novel drugs, such as finerenone, esaxerenone, sodium–glucose co-transporter-2 inhibitors, glucagon-like peptide-1 analogues and sacubitril/valsartan.

## 1. Introduction

The prevalence of hypertension and type 2 diabetes (T2D) is still increasing worldwide. The International Diabetes Federation reported that the number of cases of diabetes was estimated to be 463 million in 2019 and would increase to 700 million by 2045 [1]. In 2010, there were about 1.39 billion diagnosed cases of hypertension [2]. A global burden of disease analysis conducted in 2015 indicated that the prevalence of systolic blood pressure (BP) ≥ 140 mmHg increased from 17.3% to 20.5% between 1990 and 2015 [3].

Hypertension influences diabetes, and so diabetes affects hypertension. It has been shown that patients without controlled blood pressure despite hypotensive treatment have an increased risk of diabetes development [4]. Systolic BP may be a predictor of the development of T2D, especially in the 40 to 49 years age group, independent of obesity or the presence of peripheral vascular disease [5]. On the other hand, individuals with T2D have up to a three times higher prevalence of hypertension in comparison to their healthy counterparts [6].

Hypertension and diabetes are components of metabolic syndrome; they coexist and affect each other’s courses. Constantly elevated blood pressure occurs in 50–80% of patients suffering from T2D and in 30% of individuals with type 1 diabetes [7,8]. The coexistence of these two diseases is associated with a six-fold increased risk of cardiovascular events in comparison to healthy individuals [9]. Hypertension in patients with diabetes is associated with a 57% increased risk of any cardiovascular disease event and a 72% increased risk of all-cause death after adjustment for demographic and clinical variables [10]. In individuals with T2D and hypertension, microvascular and macrovascular complications are significantly more common than in those without hypertension [11]. Discovering the missing link between these two diseases is essential to protect this growing group of patients from unfavourable cardiovascular events. It can also aid the search for new therapies aimed at the exact cause of homeostatic failure. Today, new drugs are investigated in terms of hypotensive features in diabetic patients in order to protect them from complications as much as possible.

This review briefly describes the pathophysiology of hypertension, especially under the condition of T2D, and outlines attempts over the years to define targets for the treatment of high blood pressure in patients living with diabetes. It mentions the standard hypotensive treatment and focuses on novel drugs that have pleiotropic properties, such as finerenone, esaxerenone, sodium–glucose co-transporter-2 inhibitors, glucagon-like peptide-1 analogues and sacubitril/valsartan.

## 2. Pathophysiology of Hypertension in Diabetes

Hypertension may be divided into secondary hypertension, which has a precise known cause, for instance, renal artery stenosis, aortic coartaction, hyperthyroidism, or sleep apnea, and essential hypertension. Essential hypertension is defined as elevated BP without any known causes (after the exclusion of secondary reasons), usually clustering with aging, obesity, insulin resistance, diabetes and hyperlipidaemia—factors which are known to be cardiovascular risk factors [12]. Inappropriate control of hypertension may lead to hypertrophy of the left ventricle, damage to the kidneys manifested in microalbuminuria leading to renal failure, stroke or heart attack, cognitive dysfunction and dementia [12].

**Figure 1:**
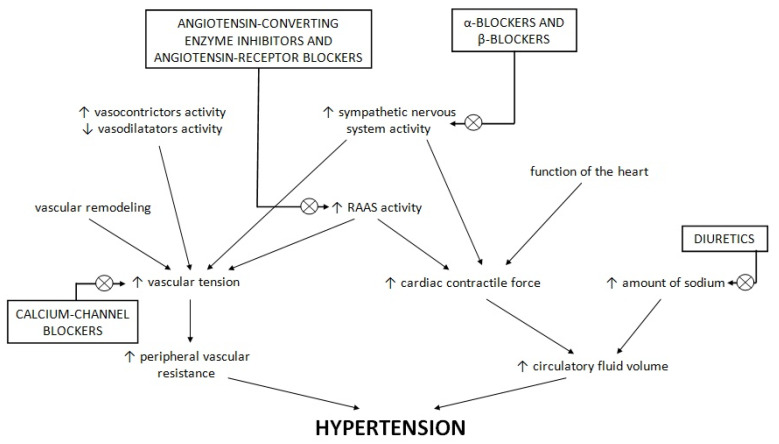
The pathogenesis of hypertension and targets for typical hypotensive drugs.

The main phenomena controlling blood pressure are peripheral vascular resistance and circulatory fluid volume. Peripheral vascular resistance results from vascular tension, which is affected by the rennin–angiotensin–aldosterone system (RAAS), other vasoconstrictors and vasodilators, the activity of the sympathetic nervous system, and vascular remodelling [13]. The overexpression of RAAS in insulin-sensitive tissues results in the impairment of metabolic signalling responses to insulin; an increased level of angiotensin II leads to decreased signalling through the phosphoinositol-3-kinase/protein kinase C [14,15,16]. Vascular remodelling and endothelial dysfunction refer to small-resistance arteries, which largely contribute to the decrease in precapillary blood pressure, and thus are largely responsible for vascular resistance [17,18]. Dysfunction of the endothelium manifests in the increased production of reactive oxygen species and reduced bioavailability of nitric oxide as a consequence [19,20]. This leads to decreased endothelium-dependent relaxation to endothelial agonists such as acetylcholine [21]. Moreover, reduced vasodilatation caused by the endothelial dysfunction hinders insulin and glucose in reaching the peripheral tissues and weakens glucose uptake stimulated by insulin [22,23]. In the Framingham Offspring Study it was proven that the von Willebrand factor antigen or plasminogen activator inhibitor-1 antigen—plasma markers indicating the dysfunction of endothelium—were connected with a higher risk of new-onset type 2 diabetes [24]. This association was independent of other risk factors of diabetes development, such as inflammation, insulin resistance and obesity [24]. Increased BP was found to enlarge the level of markers of inflammation connected with the insulin signalling pathway and function of β cells, likely participating in the development of diabetes in this way [25,26]. On the other hand, circulatory fluid volume is regulated by body fluid volume, which is mainly affected by sodium balance and cardiac contractile force, in turn influenced by the function of the heart and activity of the sympathetic nervous system and RAAS [13]. An imbalance at any stage of this homeostatic system results in hypertension. A short summary of hypertension pathogenesis and targets of typical hypotensive drugs is presented in Figure 1.

Figure 1 shows the changes in the balance between the sympathetic nervous system, RAAS, vascular tension, hearth function and sodium level. These changes lead to an increase in peripheral vascular resistance and the volume of circulatory fluid and, as a consequence, promote hypertension development. Typical hypotensive drugs are targeted towards these imbalance changes, modifying the course of hypertension. α- and β-blockers inhibit the sympathetic nervous system, and angiotensin-converting enzyme inhibitors and angiotensin receptor blockers influence RAAS activity. Calcium channel blockers affect calcium channels in the vasculature, promoting the release of vascular smooth muscles. Diuretics intensify natriuresis, which decreases the concentration of sodium.

T2D results from the functional failure of β-cells, which is triggered by insulin resistance [27]. It is characterised by hyperinsulinemia and hyperglycaemia. It has been observed that non-obese individuals without glucose tolerance impairments and with hypertension who are not treated with hypotensive drugs show hyperinsulinemia and insulin resistance [28,29]. This observation suggests that insulin resistance may play an important role in hypertensive patients. Further studies confirmed a significant relationship between the concentration of insulin and BP [30,31]. An increased level of glucose leads to increased osmolarity of the plasma and escape of water from the cells into the vasculature, resulting in increased circulatory fluid volume [32]. Moreover, hyperglycaemia also leads to an increase in glucose filtered in the glomerulus, which ultimately leads to sodium reabsorption [33,34]. Under the condition of hyperinsulinemia, sodium reabsorption is accelerated in renal tubules, also leading to hyperosmolarity [35]. In addition, hyperinsulinemia activates the sympathetic nervous system, among other processes, through the leptin-mediated activation of the pro-opiomelanocortin pathway, and stimulates the excretion of renin, which ultimately leads to a cardiac output (CO) increase and higher peripheral vascular resistance [36,37]. Insulin also promotes fat accumulation and obesity development, leading to the activation of the sympathetic nervous system, and increased CO and heart rate [32,33,34,35,36,37,38,39,40]. CO rises proportionally to the level of oxygen and perfusion demands in obesity [41].

Amylin is a peptide that is cosecreted with insulin by β-cells and participates in carbohydrate metabolism, inhibiting the incorporation of glucose into muscular glycogen and decreasing the secretion of insulin [42,43]. Under the conditions of insulin resistance and hyperinsulinemia, the concentration of this peptide is subsequently elevated [44,45,46]. It has been indicated that amylin increases the concentration of active rennin and may be responsible for RAAS activation, thus participating in the hypertension development under the condition of insulin resistance [47,48]. Moreover, RAAS may be overactivated by the factors secreted by adipose tissue that promote the release of angiotensinogen and aldosterone [49].

**Figure 2:**
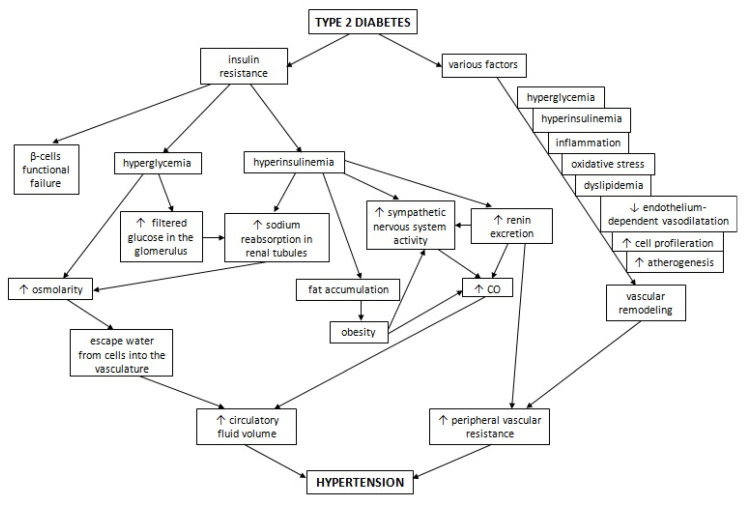
The influence of T2D on hypertension development.

Hyperglycaemia, hyperinsulinemia, inflammation and oxidative stress development, alongside the dyslipidaemia associated with T2D, contribute to vascular remodelling. This in turn causes arterial stiffness and an increase in peripheral vascular resistance, leading to the loss of blood pressure autoregulation [13]. Under physiological conditions, insulin enhances nitric oxide release and promotes vasodilatation induced by acetylcholine, but under the condition of insulin resistance, endothelium-dependent vasodilatation is reduced [50,51,52]. Moreover, insulin stimulates the growth signalling cascade via mitogen-activated protein kinase and promotes cell proliferation, whereas hyperinsulinemia may promote vascular remodelling in this way [53]. Insulin resistance and hyperinsulinemia may also accelerate the process of atherogenesis [54,55]. This combination of changes occurring in diabetes may impact the main phenomena involved in the development of hypertension. The influence of T2D on hypertension development is presented in Figure 2.

Insulin resistance is the main phenomenon underlying the pathophysiology of diabetes. Hyperglycemia and hyperinsulinemia, as well as other accompanying states, for instance inflammation, oxidative stress, dyslipidaemia or increased atherogenesis, affect the homeostatic system that regulates blood pressure. They activate the sympathetic nervous system and RAAS, trigger the remodelling of the vasculature leading to a larger peripheral vascular resistance and increases in CO and circulatory fluid volume. As a result, T2D participates in hypertension development.

## 3. Attempts to Define Targets for Treatment of High Blood Pressure in Diabetic Patients

Both hypertension and diabetes are risk factors for cardiovascular disease, leading to an increase in mortality due to coronary artery disease, heart failure or stroke [56,57]. Therefore, it is hypothesised that a reduction in BP should bring benefits for patients with T2D.

The first trial that focused on this issue was the UK Prospective Diabetes Study (UKPDS). In the study, tight blood pressure control (<150/85 mmHg) was associated with a 24% reduction in end points related to diabetes, a 32% reduction in deaths associated with diabetes, a 44% reduction in strokes, and a 37% reduction in microvascular end points, mainly due to a decreased risk of retinal photocoagulation, in comparison to the less tight control of BP (<180/105 mmHg) [58]. In the long-term follow-up from the Action to Control Cardiovascular Risk in Diabetes-Blood Pressure (ACCORD-BP) trial, a subgroup of patients with T2D and a high risk of cardiovascular diseases was analysed. After 9 years of intensive blood pressure control (systolic BP < 120 mmHg), a 25% reduction in composite cardiovascular death, nonfatal stroke and nonfatal myocardial infarction was observed, which was achieved mainly due to the reduction in nonfatal myocardial infarction [59]. Another trial, the Systolic Blood Pressure Intervention Trial (SPRINT), enrolled 9361 participants without diabetes but with systolic blood pressure ≥130 mm Hg and increased cardiovascular risk. Patients were randomised into two groups—a standard treatment group with a systolic BP target of between 135 and 140 mm Hg, and an intensive treatment group with a target of <120 mmHg. The mean systolic blood pressure of 121.4 mmHg in the intervention group was reached, compared to 136.2 mm Hg in the standard treatment group. After 3 years, a significant reduction in all-cause mortality and primary composite outcomes in the intensive treatment group was shown [60]. The post hoc analysis of SPRINT participants with prediabetes also demonstrated the beneficial effects of intensive systolic blood pressure treatment in this subgroup, supporting the statement that this effect may also be present in patients with diabetes [61]. A meta-analysis of 19 trials, including five trials enrolling patients with diabetes, suggested that intensive antihypertensive treatment diminished the risk of cardiovascular events by 14%. The reductions in stroke, myocardial infarction and albuminuria progression were statistically significant [62]. Another meta-analysis of 40 trials, including diabetic patients, shows that a reduction in systolic BP to below 130 mm Hg was associated with a lower risk of retinopathy, stroke and albuminuria [63].

The target threshold of antihypertensive treatment has been changed multiple times over the past decade in response to various guidelines. Currently, guidelines state that antihypertensive treatment should be implemented in patients with diabetes if their BP is ≥140/90 mmHg and should be sufficiently intensive to reduce it to lower than 130/80 (<140/90 in elderly patients) [64].

## 4. Standard Antihypertensive Drugs in the Therapy of Patients with Diabetes

Commonly used antihypertensive drug classes include angiotensin-converting enzyme inhibitors (ACEIs), angiotensin receptor blockers (ARBs), calcium channel blockers (CCBs), thiazide-like diuretics, mineralocorticoid receptor antagonists (MRAs), and β-blockers. Less commonly used antihypertensive treatments include α-blockers, renin inhibitors, loop diuretics, substances affecting the central nervous system such as methyldopa or clonidine, and drugs that directly lower the tension of vascular smooth muscle, for instance dihydralazine. The exact choice of hypotensive drugs depends on various factors, such as comorbidities, estimated glomerular filtration rate, side effects and ethnicity.

ACEIs and ARBs consistently and significantly reduce the incidence of T2D when used in patients suffering from hypertension or congestive heart failure, likely because of an improvement of insulin secretion and insulin sensitivity [65]. They are strongly recommended as first-line therapies in patients suffering from hypertension, diabetes and coronary artery disease, as they are proven to reduce cardiovascular events in diabetic patients [66,67,68,69]. They should be added to the therapy as early as possible in order to prevent blood vessels from remodelling [70]. Moreover, they ought to be the first option for BP control in patients with diabetes and coexisting severe albuminuria (albumin-to-creatinine ratio, ACR, >300 mg/g) and should be considered when ACR is between 30 and 299 mg/g because they lead to a reduction in the risk of kidney disease progression [71]. In the HOPE trial treatment with ramipril, one of the ACEIs, significantly reduced the risk of the composite end points, all-cause mortality, and hospitalizations caused by heart failure when used in diabetic patients with present microalbuminuria [72]. The Action in Diabetes and Vascular Disease: Preterax and Diamicron Modified-Release Controlled Evaluation (ADVANCE) trial indicated that the addition of perindopril and indapamide to therapy reduces all-cause and cardiovascular disease mortality and decreases macrovascular and microvascular outcomes in comparison to a placebo [73]. The Avoiding Cardiovascular Events through Combination Therapy in Patients Living with Systolic Hypertension (ACCOMPLISH) trial showed that therapy with an ACEI and a dihydropyridine CCB is superior to therapy with an ACE inhibitor and a thiazide diuretic in reducing adverse cardiovascular events in patients with and without diabetes; however, the dose of hydrochlorothiazide used in the trial was lower than the level shown to effectively decrease cardiovascular disease events [74,75].

MRAs, spironolactone and eplerenon, are other drugs which affect RAAS. The addition of spironolactone to standard hypotensive treatment was found to reduce the level of albuminuria in patients with diabetes complicated by diabetic nephropathy [76]. Moreover, the addition of spironolacton to a maximal dose of lisinopril resulted in greater nephroprotective properties in patients with diabetic kidney disease in comparison with the addition of losartan to the same dose of ACEI [77].

CCBs are recommended as first-line treatment in diabetic patients, especially in elderly individuals with isolated systolic hypertension [78]. Previous studies suggested that CCBs might prevent diabetes by the inhibition of β-cell apoptosis and improvement of β-cell function, but in a meta-analysis conducted by Noto et al., this hypothesis was not proven [79,80].

Therefore, ACEIs, ARBs, CCBs and thiazide-type diuretics are all acceptable options for diabetic patients as an initial hypotensive treatment. It is crucial to also consider the adverse effects of antihypertensive drugs, especially those associated with cardiometabolic consequences. Treatment with thiazide-type diuretics such as chlorthalidone may result in hyperglycaemia because of their properties that influence insulin resistance [81,82,83]. Moreover, most β-blockers are not recommended as first-line treatment in patients with diabetes because of their negative cardiometabolic effects: increasing triglyceride level, decreasing HDL cholesterol level, hiding symptoms of hypoglycaemia and impairing insulin sensitivity [84]. Moreover, it is supposed that they may also increase the risk of diabetes development, especially when used in individuals with high body weight, in comparison with an alternative substances [77]. On the contrary, not all β-blockers show such adverse effects on glucose homeostasis. Carvedilol, nebivolol, labetalol and third-generation β-blockers not only block β-adrenoreceptors, but also show additional properties promoting vasodilatation and resulting in less adverse effects on metabolism [85,86,87,88,89,90,91,92]. The Glycemic Effects in Diabetes Mellitus: Carvedilol-Metoprolol Comparison in Hypertensives (GEMINI) trial involved patients with T2D and hypertension. It compared the metabolic and glycaemic effects of treatment with metoprolol tartrate to treatment with carvedilol. The use of carvedilol did not affect glycaemic control and improved insulin sensitivity [93]. The lowest probability of triggering diabetes due to hypotensive treatment seems to occur with the use of ARBs and ACEIs, followed by CCBs [94].

## 5. Novel Antihypertensive Drugs

Aside from standard hypotensive drugs, there are many new therapeutic possibilities that show additional beneficial properties, which may be especially advantageous in patients with T2D.

Finerenone is a novel non-steroidal mineralocorticoid receptor agonist with a more selective activity than spironolactone and eplerenone. It prevents the activation of mineralocorticoid receptor by aldosterone, and thus helps to reduce remodelling, fibrosis and inflammation processes, especially in the heart, kidney and peripheral vasculature. Finerenone is mainly used in heart failure treatment, and it can cause a decrease in N-terminal pro-B-type natriuretic peptides (NT-proBNP) levels. Aside from this, it is possible to use finerenone to treat refractory hypertension and diabetic nephropathy due to its ability to reduce albuminuria. Because the activity of finerenone is more selective, it does not cause a significant increase in serum potassium level [95]. Several trials were conducted to prove the beneficial role of finerenone in the treatment of patients with T2D. The Finerenone in Reducing Kidney Failure and Disease Progression in Diabetic Kidney Disease (FIDELIO-DKD) trial enrolled 13,911 patients with chronic kidney disease and T2D, 45.9% of whom had cardiovascular disease at baseline. After a median follow-up of 2.6 years, patients treated with finerenone had a reduced risk of composite cardiovascular outcomes, including myocardial infarction, stroke, time to cardiovascular death or hospitalisation for heart failure, compared with a placebo. Additionally, renal disease progression was reduced by 18% [96]. Another trial, Finerenone in Reducing Cardiovascular Mortality and Morbidity in Diabetic Kidney Disease (FIGARO-DKD), enrolled 7347 patients with T2D and stage 1 or 2 chronic kidney disease with severely elevated albuminuria, or stage 2 to 4 chronic kidney disease with moderately elevated albuminuria. The use of finerenone decreased chronic kidney disease progression and depleted the incidence of cardiovascular events by 13% [97]. The FInerenone in chronic kiDney diseasE and type 2 diabetes: Combined FIDELIO-DKD and FIGARO-DKD Trial programme analYsis (FIDELITY) showed that the renal composite outcomes were reduced by 23%, and the cardiac composite outcomes were reduced by 14% with finerenone treatment. The mean systolic blood pressure was reduced by 3.7 mmHg at 4 months, and the effects of the drug were independent of baseline systolic BP [98].

Esaxerenone, another novel non-steroidal mineralocorticoid receptor agonist, has been already approved in Japan for the treatment of hypertension and diabetic nephropathy [99]. Due to its high potency and selectivity for mineralocorticoid receptor compared with eplerenone and spironolactone, the use of esaxerenone comes with the smaller risk of hyperkalemia, gynecomastia, amenorrhea and impotence [100]. After treatment with esaxerenone monotherapy, the reduction in sitting blood pressure from baseline to the end of treatment was −18.5/−8.8 mmHg, and after treatment as an add-on therapy to a renin-angiotensin system inhibitor the reduction was −17.8/−8.1 mmHg [101]. Moreover, a phase III clinical trial Esaxerenone (CS-3150) in Patients with Type 2 Diabetes and Microalbuminuria (ESAX-DN) demonstrated that, in patients with T2D and microalbuminuria, an addition of esaxerenone to hypertension therapy resulted in a reduced progression of albuminuria [102].

Sodium–glucose co-transporter-2 inhibitors (SGLT-2is or flozins) and glucagon-like peptide-1 analogues (GLP-1 analogues) are novel classes of antidiabetic drugs. Aside from the ability to reduce glycaemia, they possess a wide range of pleiotropic modes of action, such as cardio- and nephroprotective properties or body weight and blood pressure reduction. SGLT-2is act mainly by blocking glucose and sodium reabsorption in the proximal renal tubule, resulting in glycosuria. Increased osmotic diuresis and natriuresis leads to plasma volume depletion and, as a consequence, to blood pressure reduction. Drugs such as canagliflozin, dapagliflozin, empagliflozin or ertugliflozin belong to the group of SGLT-2is. SGLT-2is influence the mechanisms responsible for the pathogenesis of hypertension in diabetic patients. They improve arterial stiffness and endothelial dysfunction, reduce oxidative stress and preserve the circadian BP pattern [103].

The sodium hydrogen exchanger-3 (NHE-3) plays a role in the regulation of extracellular volume and blood pressure through the reabsorption of sodium in the kidney. In patients with T2D, increased levels of insulin and glucose stimulate the activity of NHE-3. Enhanced sodium influx causes a rise in peripheral vascular resistance, which increases cardiac output [104]. Due to the similar localisation of NHE-3 and SGLT-2 in the kidney, it seems possible that SGLT-2is trigger diuresis via NHE-3 inhibition [105]. They also modulate the function of the sodium hydrogen exchanger-1 (NHE-1), mainly localised in the heart and blood vessels, promoting cardiac contraction, oxidative stress reduction and a vasodilating effect [106]. The very first trial that demonstrated the cardiological benefits of empagliflozin was Empagliflozin Cardiovascular Outcome Event Trial in Type 2 Diabetes Mellitus Patients (EMPA-REG OUTCOME). The study enrolled 7020 patients with T2D who received 10 mg or 25 mg of empagliflozin or placebo once daily. After a median observation time of 3.1 years, patients treated with empagliflozin experienced a 38% reduction in the risk of cardiovascular death, and the effect was independent of the metabolic control of each group [107]. Similar trials were created with dapagliflozin (DECLARE-TIMI 58) and canagliflozin (CANVAS) and showed that the use of SGLT-2is helps to reduce the risk of hospitalization caused by heart failure [108,109]. Afterwards, the beneficial effect of flozins on heart failure treatment was also confirmed for patients without diabetes (DAPA HF trial with dapagliflozin) and with preserved and reduced ejection fractions (EMPEROR-PRESERVED and EMPEROR-Reduced with empagliflozin) [110,111,112]. Moreover, SGLT-2is led to maintenance of the renal function by a reduction in hyperfiltration and intraglomerular pressure. In the CREDENCE trial, patients with T2D and chronic kidney disease treated with canagliflozin had a reduced risk of serious renal and cardiovascular events [113]. Additionally, in the DAPA-CKD study, patients treated with dapagliflozin experienced a reduction in major adverse renal events, such as end-stage renal disease, or a 50% decline in GFR, and decrease in the risk of renal and cardiovascular death [114]. The Evaluation of Ertugliflozin Efficacy and Safety Cardiovascular Outcomes Trial (VERTIS CV) showed that patients with T2D treated with ertugliflozin also had a lower risk of first and total hospitalization for heart failure and death due to cardiovascular reasons [115]. Promisingly, the combination of finerenone and empagliflozin in preclinical hypertension-induced cardiorenal disease exhibits cardiovascular protective effects, such as a reduction in proteinuria, blood pressure, creatinine and uric acid level, histopathological cardiac and renal lesions, and mortality [116].

GLP-1 analogues act mainly by an incretin effect, stimulating insulin release, suppressing glucagon secretion, delaying gastric emptying and promoting satiety [103]. They can be divided into two groups according to their pharmacokinetics: short- and long-acting. Belonging to the short-acting GLP-1 analogues, exenatide can be taken twice per day and lixisenatide taken once daily. First of all, they reduce postprandial glucose levels and delay gastric emptying, whereas the long-acting GLP-1 analogues are liraglutide, dulaglutide, semaglutide and long-acting exenatide. They reduce mainly fasting glucose and HbA1c levels. Most GLP-1 analogues are used as subcutaneous injections, except for semaglutide, which also has an oral form [117]. Both groups of GLP-1 analogues cause body weight reduction and insulin sensitivity improvements, which are important mechanisms of BP regulation. Receptors of GLP-1 are situated in the vascular smooth muscle and endothelial cells, and their activation results in nitric oxide release. Additionally, the diuretic and natriuretic effects of GLP-1 analogues may influence blood pressure [103]. Moreover, GLP-1 analogues possess cardio- and nephroprotective properties. The Liraglutide Effect and Action in Diabetes: Evaluation of Cardiovascular Outcome Results (LEADER) trial enrolled 9340 patients with type 2 diabetes and high cardiovascular risk. Each of them received 1.8 mg of liraglutide (or the maximal tolerated dose) or placebo. After a median follow-up of 3.8 years, the risk of death from cardiovascular causes, nonfatal stroke or nonfatal myocardial infarction among patients with T2D was lower with liraglutide than with a placebo [118]. In the Trial to Evaluate Cardiovascular and Other Long-term Outcomes with Semaglutide in Subjects with Type 2 Diabetes (SUSTAIN-6) 3297 patients with T2D received once-weekly semaglutide in doses of 0.5 mg or 1 mg or a placebo for 104 weeks. The beneficial effect of semaglutide on the reduction in cardiovascular adverse effects was mainly due to the significantly lower amount of nonfatal stroke among patients receiving semaglutide compared to the placebo group [119]. The LEADER and SUSTAIN-6 trials also showed that GLP-1 analogues help to reduce the risk of diabetes nephropathy occurrence and progression by diminishing albuminuria [118,119]. The REWIND trial not only showed that patients treated with 1.5 mg of dulaglutide have lower cardiovascular risk, but also lower body weight, HbA1c, arterial pressure and cholesterol level [120], while in the PIONEER 6 trial that enrolled 3183 patients receiving 14 mg of oral semaglutide, the rate of all-cause death and cardiovascular events was significantly reduced [121]. Table 1 summarizes the effects of SGLT-2is and GLP-1 analogues on blood pressure.

Another new drug used mainly for the treatment of heart failure is a combination of valsartan, an angiotensin receptor blocker, and sacubitril, a neprilysin inhibitor. Due to the inhibition of neprilysin activity, the variety of endogenous vasoactive peptides increases, which results in enhanced natriuresis, diuresis and vasodilatation, and a reduction in cardiac fibrosis and hypertrophy [124]. A meta-analysis of five randomised controlled trials showed that treatment with sacubitril/valsartan was associated with a significant reduction in both systolic and diastolic blood pressure in elderly hypertensive patients compared with angiotensin receptor blocker use [125]. In the Prospective Comparison of ARNI With ACEI to Determine Impact on Global Mortality and Morbidity in Heart Failure (PARADIGM-HF) trial, median B-type natriuretic peptide (BNP) and NT-proBNP concentration was measured after 4 to 6 weeks, 8 to 10 weeks, and 9 months of treatment with sacubitril/valsartan. A study showed that these biomarkers can be used as a predictors of the risk of major adverse outcomes in patients treated with sacubitril/valsartan [126]. A post hoc analysis of patients with diabetes and heart failure with reduced ejection fraction enrolled in the PARADIGM-HF study revealed that individuals who received sacubitril/valsartan had a greater reduction in HbA1c concentration over at a 3-year follow-up than those receiving enalapril [127]. Sacubitril/valsartan is likely to improve glycaemic control mostly through an improvement in insulin sensitivity [128]. The Aforementioned Study to Evaluate the Effect of Dapagliflozin on the Incidence of Worsening Heart Failure or Cardiovascular Death in Patients With Chronic Heart Failure (DAPA-HF) trial enrolled 4744 patients with heart failure and reduced ejection fraction. Of these patients, 10.7% were treated with sacubitril/valsartan at baseline. The occurrence of primary endpoint (heart failure worsening or cardiovascular death) was similar in both groups treated with dapagliflozin and with or without sacubitril/valsartan. The use of both drugs together could likely decrease morbidity and mortality in patients with heart failure and reduced ejection fraction [110]. A comparison of the novel drugs is shown in Table 2.

## 6. Conclusions

Patients with diabetes and hypertension belong to a group with very high cardiovascular risk. Both diabetes and hypertension influence each other and often coexist with other components of metabolic syndrome. Thus, it is of great importance to administer treatment to these patients as early as possible that not only allows the alleviation of hypertension or hyperglycaemia, but also poses a wide range of additional beneficial modes of action. Novel non-steroidal mineralocorticoid receptor agonists, such as finerenone and esaxerenone, in addition to BP regulation, may cause a reduction in microalbuminuria and reduce the risk of cardiovascular events. SGLT-2 inhibitors and GLP-1 analogues are antidiabetic drugs with cardio- and nephroprotective properties and the ability to reduce blood pressure and body weight. Sacubitril/valsartan is a combination used in heart failure treatment that also has the ability to reduce blood pressure and improve glucose tolerance. Further studies are needed to explain the exact mechanisms of the multifarious actions of these medications, but their application should be considered from the very beginning of treatment in order to better protect patients from the consequences of their diseases.

## Figures and Tables

**Table 1:** Effects of specific SGLT-2 inhibitors and GLP-1 analogues on blood pressure [107,109,116,118,119,120,122,123].

	Systolic Blood Pressure	Diastolic Blood Pressure
Empagliflozin 25 mg/d	−4.78 mmHg	−1.90 mmHg
Canagliflozin	−3.93 mmHg	−1.39 mmHg
Dapagliflozin	−2.70 mmHg	−0.70 mmHg
Exenatide	−1.57 mmHg	+0.25 mmHg
Liraglutide	−1.20 mmHg	+0.60 mmHg
Dulaglutide	−1.70 mmHg	+0.12 mmHg
Semaglutide	−2.60 mmHg	+0.14 mmHg

**Table 2:** Comparison of novel antihypertensive drugs [52,129,130,131,132,133,134].

Name of Drug	Mode of Action	Dosage	Method and Route of Administration	Indications	Contraindications	Side Effects
Finerenone	non-steroidal MRA	10–20 mg	Oral use once daily	Diabetic kidney disease Heart failure	Hyperkalaemia Kidney failure Addison disease	Increased level of serum potassium
Esaxerenone	non-steroidal MRA	1.25–5 mg	Oral use once daily	Hypertension Diabetic nephropathies	Hyperkalaemia	Increased level of serum potassium Hyperuricemia
Canagliflozin	SGLT-2i	100–300 mg	Oral use once daily	Type 2 diabetes	Kidney failure Ketoacidosis Hospitalization Hypotension	Hypoglycaemia Candidiasis Genito-urinary tract infection
Dapagliflozin	SGLT-2i	5–10 mg	Oral use once daily	Type 2 diabetes Chronic heart failure	Kidney failure Hypotension Liver failure Ketoacidosis	Hypoglycaemia Dizziness Dysuria Genito-urinary tract infection
Empagliflozin	SGLT-2i	10–25 mg	Oral use once daily	Type 2 diabetes Chronic heart failure	Ketoacidosis Kidney failure Liver failure	Hypoglycaemia Dehydration Genito-urinary tract infection
Exenatide	Short-acting GLP-1 analogue	5–10 µg	Subcutaneous injection twice daily	Type 2 diabetes	Type 1 diabetes and ketoacidosis Allergy and anaphylaxis Pregnancy and breast feeding Kidney failure Gastroparesis	Nausea Vomiting
Lixisenatide	Short-acting GLP-1 analogue	10–20 µg	Subcutaneous injection once daily	Type 2 diabetes	Pancreatitis Kidney failure Dehydration	Hypoglycaemia Nausea Vomiting Diarrhoea Headache
Dulaglutide	Long-acting GLP-1 analogue	0.75–1.5 mg	Subcutaneous injection once weekly	Type 2 diabetes	Type 1 diabetes and ketoacidosis End-stage renal disease Dehydration Gastroparesis Acute pancreatitis	Hypoglycaemia Nausea Vomiting Diarrhoea Stomach ache
Long-acting exenatide	Long-acting GLP-1 analogue	2 mg	Subcutaneous injection once weekly	Type 2 diabetes	Type 1 diabetes and ketoacidosis Allergy and anaphylaxis Pregnancy and breast feeding Kidney failure Gastroparesis	Nausea Vomiting
Liraglutide	Long-acting GLP-1 analogue	0.6–1.8 mg	Subcutaneous injection once daily	Type 2 diabetes Obesity and overweight with additional metabolic disease	Congestive heart failure Pancreatitis Dehydration Thyroid diseases Gastroparesis	Nausea Vomiting Diarrhoea
Semaglutide	Long-acting GLP-1 analogue	(0.25–1.0 mg)/(3–14 mg)	Subcutaneous injection once weekly/oral use once daily	Type 2 diabetes	Congestive heart failure State after bariatric operation Acute pancreatitis	Hypoglycaemia Nausea Diarrhoea
Sacubitril/valsartan	ARB and neprilysin inhibitor	(24 mg/26 mg)-(97 mg/103 mg)	Oral use twice daily	Chronic heart failure with reduced ejection fraction	Kidney failure Hyperkalaemia Liver failure Allergy and anaphylaxis Hypotension	Hyperkalaemia Hypotension Kidney function disorder

## Data Availability

Not applicable.
